# Commemorating a conservation trifecta: Three new species of *Glossoloma* (Gesneriaceae) honoring a donor, a family of forest stewards, and a conservation visionary

**DOI:** 10.3897/phytokeys.271.181141

**Published:** 2026-03-04

**Authors:** John L. Clark

**Affiliations:** 1 Marie Selby Botanical Gardens, 1534 Mound St., Sarasota, FL 34236 USA Marie Selby Botanical Gardens Sarasota United States of America

**Keywords:** Andes, biodiversity, Colombia, Ecuador, nomadic climber, Pastaza, taxonomy

## Abstract

During a 2016 field course in the Cerro Candelaria Reserve in the Pastaza Valley on the eastern slopes of the Ecuadorian Andes, three new species of *Glossoloma* (Gesneriaceae) were discovered along a single trail in one day. All three species are nomadic climbers, contrasting with the more typical subshrub habit in the genus. They are named in honor of three stakeholders from diverse backgrounds who collaborated to conserve one of the last remnants of Andean forests in the Pastaza Valley. The specific epithets commemorate their contributions to protecting these rapidly disappearing forests. The new species are *Glossoloma jostii* J.L.Clark, **sp. nov**., *G. puroanum* J.L.Clark, **sp. nov**., and *G. recaldeorum* J.L.Clark, **sp. nov**.

## Abstract (de)

Durante un curso de campo realizado en 2016 en la Reserva Cerro Candelaria, en el valle del río Pastaza, en las estribaciones orientales de los Andes ecuatorianos, se descubrieron tres especies nuevas de *Glossoloma* (Gesneriaceae) a lo largo de un solo sendero, todas en el transcurso de un solo día. Las tres especies comparten un hábito trepador nómada, en contraste con el hábito de subarbusto más típico del género. Fueron nombradas en honor a tres colaboradores de diversos orígenes que trabajaron conjuntamente para conservar uno de los últimos remanentes de bosques andinos en el valle del Pastaza. Los epítetos específicos conmemoran sus contribuciones a la protección de estos bosques que están desapareciendo rápidamente. Las nuevas especies son *Glossoloma jostii* J.L.Clark, **sp. nov**., *G. puroanum* J.L.Clark, **sp. nov**. y *G. recaldeorum* J.L.Clark, **sp. nov**.

## Introduction

The genus *Glossoloma* Hanst. is a member of the Columneinae, a Neotropical subtribe that represents approximately 16% of the total species diversity in the Gesneriaceae with approximately 525 species as of 2025 (Weber et al. 2013, 2020; Clark et al. 2020; GRC 2025). Molecular phylogenetic studies strongly support the monophyly of *Glossoloma* and further indicate that it shares a recent common ancestor with other genera that are especially species rich in the northern Andes, including *Alloplectus* Mart., *Columnea* L., and *Drymonia* Mart. (Clark and Zimmer 2003; Clark et al. 2006; Serrano-Serrano et al. 2017).

*Glossoloma* occurs from Mexico to Bolivia, with highest diversity in the northern Andes of Colombia and Ecuador. The most recent monographic revision of *Glossoloma* (Clark 2009) recognized 27 species. Four additional species have since been described (Rodas and Clark 2014; Clark and Tobar 2021; Hoyos et al. 2023; Solano-C et al. 2025), bringing the total to 31 prior to this study. The three species described here increase the number to 34 *Glossoloma* species.

*Glossoloma* is distinguished from related genera by resupinate (upside-down) flowers. The only exception is *G. anomalum* J.L. Clark, which is supported as an autapomorphic reversal to non-resupinate flowers (Clark et al. 2006). Most species in the genus are unbranched terrestrial subshrubs with erect shoots, and less frequently scandent subshrubs that are often characterized as epiphytic. The species treated here have a scandent growth form best described as a “nomadic vine” (Moffett 2000), in which plants germinate terrestrially, climb nearby vegetation by their scandent stems or adventitious roots, and may shed older basal portions as they ascend (Zotz 2013). The only other *Glossoloma* species in Ecuador that is also a nomadic climber is *Glossoloma wiehleri* J.L. Clark & Tobar, which is endemic (only known from three populations) in the Pichincha province on the western slopes of the Ecuadorian Andes (Clark and Tobar 2021). Because the nomadic habit is rare in *Glossoloma*, *G. wiehleri* is included in Table 1 for comparison with the newly described species.

**Table 1.** Geographic distribution (Ecuadorian provinces in parentheses) and diagnostic morphological characters differentiating *Glossoloma jostii*, *G. puroanum*, *G. recaldeorum*, and *G. wiehleri*.

	*Glossoloma jostii* J.L.Clark	*Glossoloma puroanum* J.L.Clark	*Glossoloma recaldeorum* J.L.Clark	*Glossoloma wiehleri* J.L.Clark & Tobar
**Leaf shape**	oblong	oblong	ovate	oblong
**Leaf texture**	leaves brittle when dry	leaves chartaceous (not brittle when dry)	leaves chartaceous (not brittle when dry)	leaves coriaceous (not brittle when dry)
**Leaf size**	5–10 × 1.5–3 cm	15–20 × 4.5–7.0 cm	3–4 × 1.5–2.0 cm	9.3–12.5 × 5–6.7 cm
**Leaf indumentum**	hispid	sericeous	pilose	velutinous
**Inflorescence bracts**	absent	present	absent	present
**Flowers per axil**	single	multiple	single	multiple
**Calyx lobes**	valvate or separate	conduplicate	conduplicate	conduplicate
**Corolla shape**	ampliate and apically constricted	nearly tubular and not apically constricted	ampliate and not apically constricted	ampliate and not apically constricted
**Corolla compression**	laterally compressed	laterally compressed	rounded (not laterally compressed)	rounded (not laterally compressed)
**Corolla surface**	smooth	ridged or ribbed	smooth	smooth
**Distribution**	eastern Andean slopes – Pastaza Valley (Tungurahua)	eastern Andean slopes – Pastaza Valley (Tungurahua)	eastern Andean slopes – Pastaza Valley (Tungurahua)	western Andean slopes of northern Ecuador (Pichincha)

The monographic revision of *Glossoloma* (Clark 2009) was based on extensive field work and herbarium research which included examination of specimens (including all types of species in *Glossoloma*) from more than 40 herbaria through extended loans and visits that spanned more than a decade of extensive field work between 1996 and 2008. The most common habit in *Glossoloma*, and also the most challenging for species circumscription, is that of unbranched terrestrial subshrubs, particularly in the absence of flowers. During the monographic revision of Glossoloma (Clark 2009), nearly all then-known species exhibited this habit. The three species described here are characterized by the less common scandent habit. The scandent habit makes the four *Glossoloma* species from Ecuador (Table 1) relatively distinctive from most congeners. Remarkably, all three were discovered along a single trail during one day of fieldwork in the Pastaza Valley along the eastern Andean slopes of the Tungurahua province.

The area from where these species were collected represents now only a fragment of a once extensive Andean forest, much of which has been lost since the 1970s due to agricultural expansion. Its continued existence is due to the vision of one family, the leadership of a conservationist, and the support of a donor. Thus, this research commemorates the conservation trifecta that saved the forest through the efforts of the EcoMinga Foundation’s Cerro Candelaria Reserve.

The upper Rio Pastaza cuts a deep straight east-facing valley 40 km long through several closely spaced parallel north-south mountain ranges bordering the western Amazon basin. Moist prevailing winds from the Amazon basin flow up this valley and successively encounter each of these ranges, dropping the most moisture on the first range facing the Amazon, and successively less moisture on each line to the west. Cerro Candelaria (3860 m) is in the second line of mountains facing the Amazon Basin.

Although sympatric speciation is documented in the literature (e.g., Stebbins 1950; Savolainen et al. 2006; Anacker and Strauss 2014), it is uncommon at such fine spatial scales. What makes this example particularly notable is not simply the occurrence of sympatric congeners, but the discovery of three newly described species along a single trail. Although remarkable, this pattern is not unprecedented in the forests where these species are presumably endemic. In 2000, the genus *Teagueia* Luer (Orchidaceae) included only six species and only one known from the Río Pastaza Valley. Within a relatively short time, Jost and his collaborators discovered and described ten additional species, all in the upper Río Pastaza Valley and all sharing distinctive floral and vegetative characteristics not present in the six previously described members of *Teagueia* (Luer 2000; Jost and Shepard 2011, 2017). In addition to Jost’s taxonomic contributions, he has discussed the Pastaza Valley as an area of unique sympatric speciation (Jost 2004), supported by ongoing research indicating that the total number of orchid species will likely exceed those currently described. Other sympatric congeners in the upper Río Pastaza watershed include four new species of *Sciodaphyllum* (formerly *Schefflera*) P. Browne (Araliaceae) discovered during a single short expedition through Fundación EcoMinga’s Río Zuñac Reserve (Neill et al. 2021) and three new *Meriania* Sw. species (Melastomataceae) from the same trail (Ulloa Ulloa et al. 2007; Fernández-Fernández et al. 2020). Thus, while the sympatric occurrence of the three new *Glossoloma* species described here is extraordinary, it is not unique within this region.

## Materials and methods

The type specimens for the three species were collected from the Cerro Candelaria reserve that is owned and operated by the EcoMinga Foundation based in the Tungurahua province in the upper Pastaza Valley along the eastern Andean slopes in Ecuador. All three species are sympatric and occur along a single trail between 2,200 and 2,660 m elevation. Each taxon is represented by a single collection from one individual, with several duplicates prepared from that same plant. Although only a single collection was made per species, repeated field observations along the trail confirmed consistent morphology and the absence of intermediate forms. The same GPS coordinate was recorded for all three collections to emphasize their sympatric distribution along the 440 m elevational gradient. To minimize disturbance, only limited cuttings were taken from one individual per species to ensure that documentation did not affect the abundance or integrity of the populations.

Extensive herbarium research from the recent monographic revision of *Glossoloma* (Clark 2009) included more than 40 herbaria with extensive loans, including type specimens for all currently known *Glossoloma*. The character states and descriptions in this study are based on Clark (2009), Beentje (2016), and Harris and Harris (2006). Digital images of live specimens were taken in the field using a Nikon DSLR with a Nikon 105 mm lens and a Nikon SB-29s ring flash. Morphological observations and measurements were made from live collections, herbarium specimens, alcohol-preserved material, and digital images; the latter were analyzed using the software program ImageJ (Schneider et al. 2012).

## Taxonomic treatment

### Glossoloma jostii

Taxon classificationPlantaeLamialesGesneriaceae

J.L.Clark sp. nov.

2B3F9391-A9EA-53B8-85FC-5DB0C916B293

urn:lsid:ipni.org:names:77377380-1

Fig. 1

#### Diagnosis.

Differs from all other congeners by its scandent subwoody habit (vs. the more common terrestrial unbranched shrub habit), brittle leaf blades (vs. chartaceous in most other congeners), apically constricted corolla (vs. non-ampliate or broad as in *G. subglabrum*), uniformly red corollas (vs. uniformly yellow, or yellow with red lobes), and valvate calyx lobes (vs. conduplicate calyx lobes where each lobe is appressed to an adjacent lobe and folded lengthwise in *G. puroanum* and *G. recaldeorum*).

**Figure 1.**
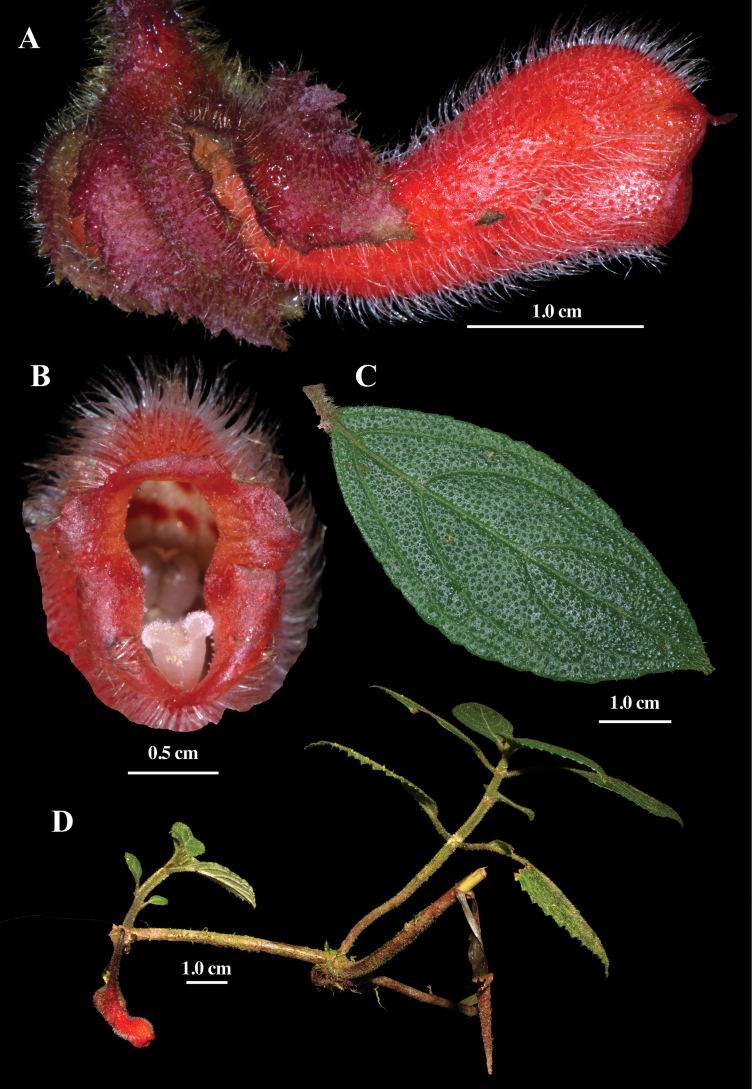
Field images of *Glossoloma jostii* J.L.Clark. **A**. Lateral view of flower; **B**. Front view of corolla; **C**. Adaxial leaf surface; **D**. Habit (**A–D**. From *J.L. Clark et al. 14803*). Photos by J.L. Clark.

#### Type.

Ecuador • Tungurahua: Cantón Baños, parroquia Río Verde, Bosque Protector Cerro Candelaria (Fundación EcoMinga), upper Pastaza watershed, high ridgeline above canyon, trail between Cerro Candelaria permanent plot #1 (2200 m) and high camp (2600 m), 1°26'26.7"S, 78°18'11.4"W, 2200–2664 m, 9 March 2016, *J.L. Clark, J.A. Mayr & D.A. Neill 14803* (holotype: QCA!; isotypes: E!, ECUAMZ!, G!, MO!, NY!, SEL! [barcode SEL089267], US!).

#### Description.

Elongate scandent nomadic climbers. ***Stems*** elongate and subwoody, terete in cross section, 3–6 mm in diameter, sparsely to densely pilose. ***Leaves*** opposite, equal in a pair; petioles 1–3 cm long, green, terete in cross-section, densely pilose; blade brittle (especially when dry), oblong, 5–10 × 1.5–3 cm, coriaceous, adaxially and abaxially green, apex acute to acuminate, base acute, margin serrate, 4–6 pairs of secondary veins, hispid abaxially and adaxially. ***Inflorescences*** reduced to a single axillary flower. ***Flowers*** resupinate, with elongate pendent pedicels, 1.6–2.2 cm long. Calyx lobes uniformly dark red, pilose on the outside and glabrous on the inside, lobes 5, nearly free, fused at the base for 2–4 mm, valvate to separate, and clasping corolla tube, lower lobe relatively smaller and narrow, ca. 1.3 × 0.4 cm, the other four lobes broadly ovate, apex acute, margins serrate, ca. 1.5 × 1.3 cm. Corolla tube ampliate on upper surface and apically constricted, appearing perpendicular relative to calyx, 2.7–3.8 cm long, gibbous at base, appearing laterally compressed, 2.0–3.5 mm wide, outside uniformly pilose, inside mostly glabrous with minute glandular trichomes apically, throat elliptic in cross section, lobes 5, subequal, margins entire to serrulate, lobes reflexed, 8–11 × 9–12 mm, uniformly red. ***Androecium*** of 4 didynamous stamens, included, filaments broad and flat, ca. 3.0 cm long, adnate to the corolla tube base for 4 mm, white, glabrous; anthers oblong, coherent by the lateral walls, dehiscing longitudinally, 4.2–6.0 × 0.7–2.0 mm. ***Gynoecium*** with a single bilobed dorsal gland; ovary superior, 4.0–5.0 × 4.0–5.0 mm, cone-shaped, puberulent; style stout, included, 3.0 cm long; stigma stomatomorphic. ***Fruit*** not observed.

#### Phenology.

Collected with flowers in March.

#### Etymology.

The specific epithet honors Lou Jost, botanist, conservationist, self-taught mathematical biologist, and co-founder of the EcoMinga Foundation. His efforts have been instrumental in protecting montane forests throughout Ecuador, particularly in the Pastaza Valley on the eastern slopes of the Andes. Jost has contributed extensively to the discovery and description of plant species, especially orchids, and his vision and leadership have been pivotal in conserving biodiversity hotspots in the region. This epithet commemorates his dedication to science, conservation, and the protection of remnant Andean forests, including the locality of the three new species described here.

#### Distribution.

*Glossoloma jostii* is currently only known from the Cerro Candelaria reserve near Río Verde in Bosque Protector Cerro Candelaria, a private reserve managed by Fundación EcoMinga on the eastern slopes of the Ecuadorian Andes.

#### Comments.

The combination of uniformly red corollas with a nomadic scandent habit is rare in *Glossoloma*. An undescribed western Andean species also shares the scandent habit and uniformly red corollas of *G. jostii*, but differs by its green calyx lobes (vs. red in *G. jostii*) and a uniformly bright yellow throat (vs. the uniformly red inner and outer surfaces in *G. jostii*; Fig. 1B). Some other species of *Glossoloma* also have uniformly red corollas, but they are readily distinguished from *G. jostii* by their unbranched, terrestrial subshrub habit and elongate tubular corollas without an apical constriction (vs. ampliated corollas that are apically constricted in *G. jostii*; Fig. 1A). Examples of unbranched terrestrial subshrub species with red corolla include *G. baguense* (L.E. Skog) J.L. Clark, *G. subglabrum* J.L. Clark, *G. oblongicalyx* (J.L. Clark & L.E. Skog) J.L. Clark, and *G. panamense* (C.V. Morton) J.L. Clark. One example of a terrestrial species of *Glossoloma* characterized with uniformly red corollas and apically constricted corolla tubes is *Glossoloma ichthyoderma* (Hanst.) J.L. Clark. *Glossoloma jostii* can be distinguished from *G. ichthyoderma* by its scandent, branched shoots (Fig. 1D). In contrast, *G. ichthyoderma* consistently has erect, unbranched shoots, with a terrestrial or epiphytic habit. Vegetatively, *G. jostii* is differentiated by brittle leaves with a hispid indumentum (Fig. 1C & Table 1). The leaves in *G. puroanum* are chartaceous (not brittle) with a sericeous indumentum on the leaf (vs. hispid indumentum in *G. jostii*; Table 1).

### Glossoloma puroanum

Taxon classificationPlantaeLamialesGesneriaceae

J.L.Clark sp. nov.

5F0725EE-E1E0-5852-AA62-CA3F6FBDCD91

urn:lsid:ipni.org:names:77377381-1

Fig. 2

#### Diagnosis.

Differs from all other congeners by the presence of a scandent subwoody habit (vs. terrestrial unbranched shrub), chartaceous leaf blades (vs. brittle leaf blades in *G. jostii*), presence of inflorescence bracts (vs. absence of inflorescence bracts in *G. jostii*), nearly tubular corolla that lacks an apical constriction (vs. ampliate and apically constricted corolla in *G. jostii*), a corolla tube with prominent longitudinal ridges (vs. smooth corolla in *G. jostii*), and cucullate calyx lobes (vs. valvate calyx lobes in *G. jostii*).

**Figure 2.**
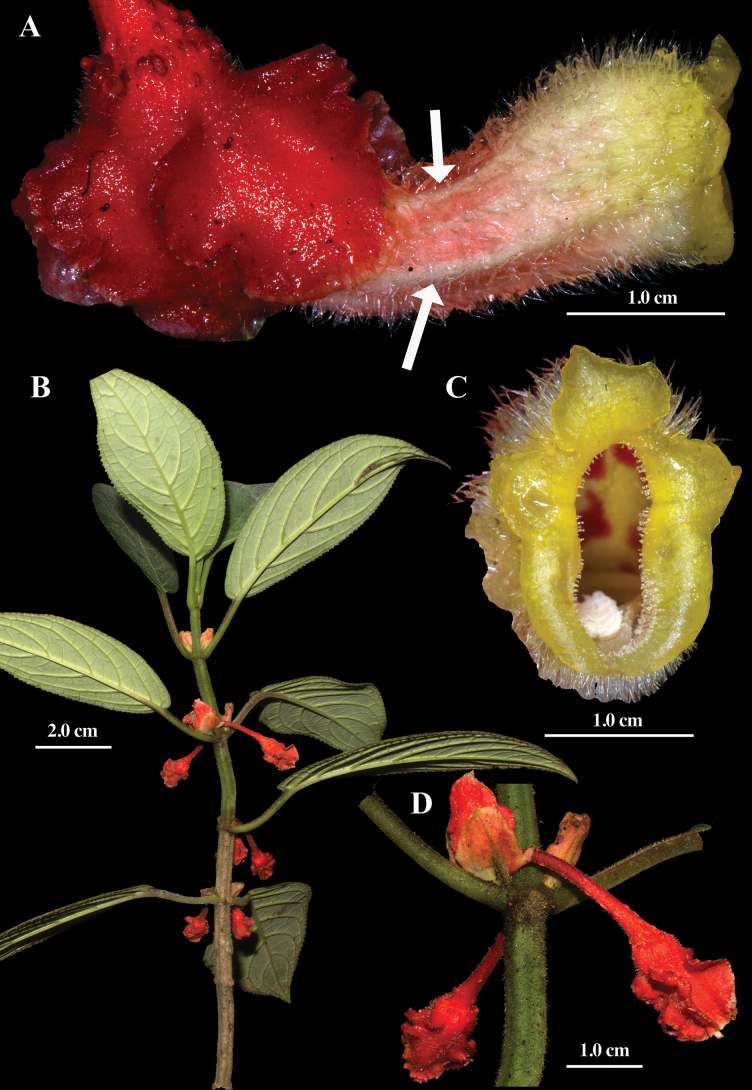
Field images of *Glossoloma puroanum* J.L.Clark. **A**. Lateral view of flower with arrows indicating the longitudinal ridges; **B**. Habit; **C**. Front view of corolla; **D**. Shoot (**A–D**. From *J.L. Clark et al. 14812*). Photos by J.L. Clark.

#### Type.

Ecuador • Tungurahua: Cantón Baños, parroquia Río Verde, Bosque Protector Cerro Candelaria (Fundación EcoMinga), upper Pastaza watershed, high ridgeline above canyon, trail between Cerro Candelaria permanent plot #1 (2200 m) and high camp (2600 m), 1°26'26.7"S, 78°18'11.4"W, 2200–2664 m, 9 March 2016, *J.L. Clark, J.A. Mayr & D.A. Neill 14812* (holotype: QCA!; isotypes: ECUAMZ!, G!, MO!, NY!, SEL! [barcode SEL089280], US!).

#### Description.

Elongate scandent nomadic climbers. ***Stems*** elongate and quadrangular in cross section, 3–6 mm in diameter, sparsely pilose. ***Leaves*** opposite, equal in a pair; petiole 3–5.5 cm long, green, terete in cross-section, sparsely pilose; blade oblong, 15–20 × 4.5–7.0 cm, chartaceous, adaxially dark green, abaxially light green, apex acute to acuminate, base rounded, margin serrate, 5–7 pairs of secondary veins, abaxially and adaxially sericeous. ***Inflorescences*** reduced to 1 to 3 axillary flowers. ***Flowers*** resupinate, with elongate pendent pedicels, 2.8–3.7 cm long. Calyx uniformly red, sparsely pilose on the outside and glabrous on the inside, lobes 5, fused at the base for 2–4 mm, conduplicate with each lobe appressed to an adjacent lobe and folded lengthwise with the margin curved inward, clasping corolla tube, lower lobe relatively smaller and narrow, ca. 1.8 × 0.8 cm, the other four lobes broadly oblong, apex rounded, margins serrate, ca. 2.0 × 1.4 cm. Corolla tube ampliate on upper surface and slightly constricted apically, appearing perpendicular relative to calyx, 3.0–4.0 cm long, gibbous at base, with prominent longitudinal ridges, appearing laterally compressed, 2.5–4.0 mm wide, outside uniformly pilose, inside mostly glabrous with minute glandular trichomes apically, throat elliptic in cross section, lobes 5, subequal, margins entire to serrulate, lobes reflexed, 8–11 × 9–12 mm, tube yellow, basally suffused with red. ***Androecium*** of 4 didynamous stamens, included, filaments broad and flat, ca. 3.8 cm long, adnate to the corolla tube base for 4 mm, white, glabrous; anthers oblong, coherent by the lateral walls, dehiscing longitudinally, 4.3–6.2 × 0.8–2.2 mm. ***Gynoecium*** with a single bilobed dorsal gland; ovary superior, 4.2–5.2 × 4.2–5.2 mm, cone-shaped, pilose; style stout, included, 3.5 cm long; stigma stomatomorphic. ***Fruit*** not observed.

#### Phenology.

Collected with flowers in March.

#### Etymology.

The specific epithet honors Puro Coffee (UK), founded by Andy Orchard, whose support through the World Land Trust was instrumental in establishing Fundación EcoMinga’s Cerro Candelaria Reserve that safeguards all three species described here. In addition, Puro Coffee, through continued donations to the World Land Trust, supports the salary of a park guard at the reserve.

#### Distribution.

*Glossoloma puroanum* is currently only known from the Cerro Candelaria reserve near Río Verde in Bosque Protector Cerro Candelaria, a private reserve managed by Fundación EcoMinga on the eastern slopes of the Ecuadorian Andes.

#### Comments.

*Glossoloma puroanum* is characterized by elongate tubular corollas with prominent longitudinal ridges (Fig. 2A). The corolla is yellow, suffused with red at the base externally, and marked internally with prominent red spots (Fig. 2C). The calyx lobes are red and folded in a conduplicate manner (i.e., each lobe appressed to an adjacent lobe and folded lengthwise with the margin curved inward – Fig. 2 A&D). Yellow corollas are common in *Glossoloma*, but this is the only species in the genus known to have both a scandent habit and corollas yellow suffused with red (Fig. 2). In addition, while most species of *Glossoloma* have terete stems, *G. puroanum* is distinctive in having subquadrangular stems (Fig. 2 B&D). The leaf blades in *G. puroanum* are chartaceous with a sericeous indumentum (Table 1). In contrast, the leaves in *G. jostii* are stiff, brittle (especially when dry) and have a hispid indumentum (Table 1). Another diagnostic character of *G. puroanum* is the presence of multiple flowers in the leaf axils (vs. single axillary flowers in *G. jostii* and *G. recaldeorum*) and presence of inflorescence bracts (vs. absence of inflorescence bracts in *G. jostii* and *G. recaldeorum*).

### Glossoloma recaldeorum

Taxon classificationPlantaeLamialesGesneriaceae

J.L.Clark sp. nov.

FC3B7CDB-EFD7-56A1-A861-107725B59350

urn:lsid:ipni.org:names:77377382-1

Fig. 3

#### Diagnosis.

Differs from all other congeners by the presence of a scandent subwoody habit (vs. terrestrial unbranched shrub), small ovate leaves (vs. oblong leaves in *G. jostii* and *G. puroanum*), a corolla tube that is rounded at the throat (vs. laterally compressed corolla tubes in *G. jostii* and *G. puroanum*), and single axillary flowers (vs. multiple flowers per leaf axil in *G. jostii* and *G. puroanum*).

**Figure 3.**
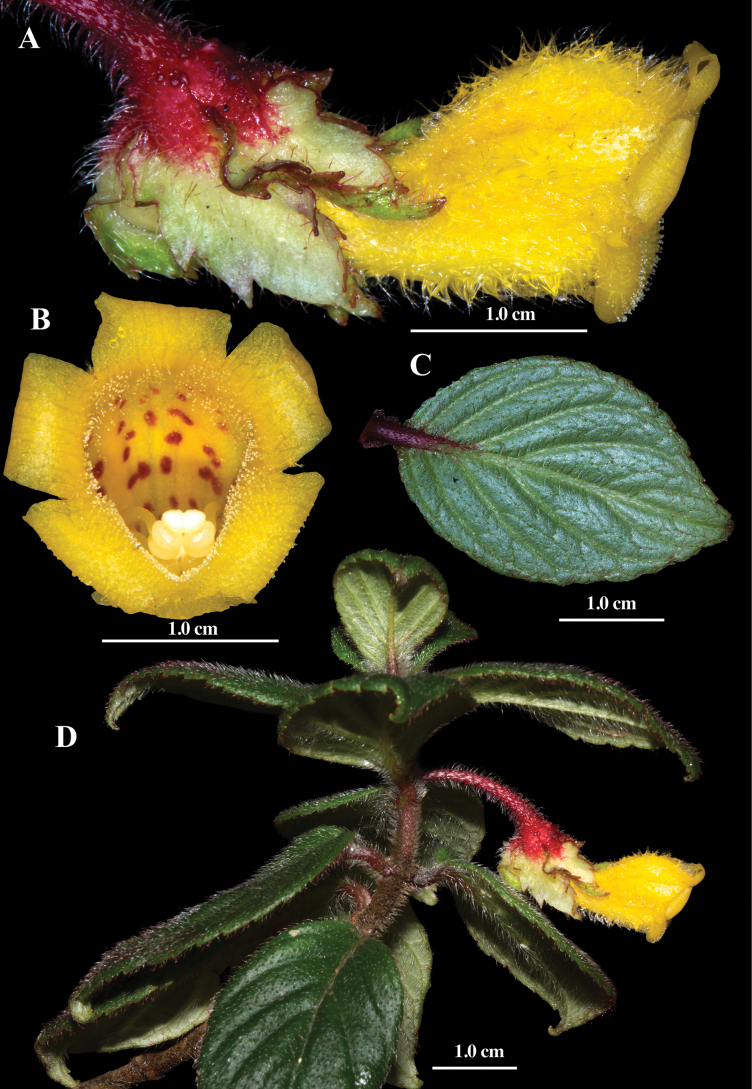
Field images of *Glossoloma recaldeorum* J.L.Clark. **A**. Lateral view of flower; **B**. Front view of corolla; **C**. Abaxial leaf surface; **D**. Habit (**A–D**. From *J.L. Clark et al. 14818*). Photos by J.L. Clark.

#### Type.

Ecuador • Tungurahua: Canton Baños, parroquia Río Verde, Bosque Protector Cerro Candelaria (Fundación EcoMinga), upper Pastaza watershed, high ridgeline above canyon, trail between Cerro Candelaria permanent plot #1 (2200 m) and high camp (2600 m), 1°26'26.7"S, 78°18'11.4"W, 2200–2664 m, 9 March 2016, *J.L. Clark, J.A. Mayr & D.A. Neill 14818* (holotype: QCA!; isotypes: CAS!, E!, ECUAMZ!, G!, MO!, NY! SEL!, US!).

#### Description.

Elongate scandent nomadic climbers. ***Stems*** elongate and terete in cross section, 2–4 mm in diameter, sparsely pilose. ***Leaves*** opposite, equal in a pair; petiole 4–10 mm long, red, terete in cross-section, sparsely pilose; blade ovate, 3–4 × 1.5–2.0 cm, coriaceous, adaxially dark green, abaxially light green, apex acute, base rounded, margin sparsely serrate, 3–5 pairs of secondary veins, abaxially and adaxially pilose. ***Inflorescences*** reduced to a single axillary flower. ***Flowers*** resupinate, with elongate pendent to horizontal 2.5–3.3 cm long pedicels. Calyx mostly light green with reddish base, sparsely pilose on the outside and glabrous on the inside, lobes 5, fused at the base for 2–4 mm, conduplicate with each lobe appressed to an adjacent lobe and folded lengthwise with the margin curved inward, clasping corolla tube, lower lobe relatively smaller and narrow, ca. 1.0 × 0.4 cm, the other four lobes broadly oblong, apex acute, margins serrate, ca. 1.3 × 0.8 cm. Corolla tube ampliate on upper surface and slightly constricted apically, appearing perpendicular relative to calyx, 2.5–3.2 cm long, gibbous at base, 2.5–4.0 mm wide, outside uniformly pilose, inside mostly glabrous with minute glandular trichomes apically, throat rounded in cross section, lobes 5, subequal, margins entire to serrulate, lobes reflexed, 8–11 × 9–12 mm, uniformly yellow. ***Androecium*** of 4 didynamous stamens, included, filaments broad and flat, ca. 3.1 cm long, adnate to the corolla tube base for 3 mm, white, glabrous; anthers oblong, coherent by the lateral walls, dehiscing longitudinally, 3.9–5.7 × 0.6–1.8 mm. ***Gynoecium*** with a single bilobed dorsal gland; ovary superior, 3.9–4.9 × 3.0–4.0 mm, cone-shaped, puberulent; style stout, included, 3.0 cm long; stigma stomatomorphic. ***Fruit*** not observed.

#### Phenology.

Collected with flowers in March.

#### Etymology.

The specific epithet honors the Recalde family of El Placer, the nearest community to the location of the species described here. Over the years, the brothers Luis, Fausto, Jesús, and Abdón Recalde, and later their children Darwin, Santiago, and Diana Recalde, have served as park guards protecting Cerro Candelaria Reserve, and have been instrumental in promoting a positive attitude in communities towards promoting forest conservation. Their keen passion for nature and their extraordinary observations have led to the discovery of many new species, especially frogs.

#### Distribution.

*Glossoloma recaldeorum* is currently only known from the Cerro Candelaria reserve near Río Verde in Bosque Protector Cerro Candelaria, a private reserve managed by Fundación EcoMinga on the eastern slopes of the Ecuadorian Andes.

#### Comments.

*Glossoloma recaldeorum* is distinguished by its small ovate leaves (Fig. 3C), the smallest currently known in the genus. The petioles are typically red, in contrast to the green petioles of congeners. The corollas are yellow, as in *G. puroanum*, but differ in being more rounded at the throat (Fig. 3B) rather than laterally compressed (Fig. 2C). In addition, the presence of solitary axillary flowers (Fig. 3D) is unique within the genus, where most species bear multiple flowers per leaf axil (e.g., Fig. 2D).

## Supplementary Material

XML Treatment for Glossoloma jostii

XML Treatment for Glossoloma puroanum

XML Treatment for Glossoloma recaldeorum

## Additional information

### Use of AI

No use of AI was reported.

### Author ORCIDs

John L. Clark https://orcid.org/0000-0002-1414-6380

### Data availability

All of the data that support the findings of this study are available in the main text.
